# A Novel Score for Predicting Alzheimer’s Disease Risk from Late Life Psychopathological and Health Risk Factors

**DOI:** 10.3390/ijerph18041802

**Published:** 2021-02-12

**Authors:** Javier Santabárbara, Juan Bueno-Notivol, Darren M. Lipnicki, Concepción de la Cámara, Raúl López-Antón, Antonio Lobo, Patricia Gracia-García

**Affiliations:** 1 Department of Preventive Medicine and Public Health, Universidad de Zaragoza, 50001 Zaragoza, Spain; jsantabarbara@unizar.es; 2 Instituto de Investigación Sanitaria de Aragón (IIS Aragón), 50001 Zaragoza, Spain; conchidlc@hotmail.com (C.d.l.C.); rlanton@unizar.es (R.L.-A.); alobo@unizar.es (A.L.); pgraciag@salud.aragon.es (P.G.-G.); 3 Centro de Investigación Biomédica en Red de Salud Mental (CIBERSAM), Ministry of Science and Innovation, 28029 Madrid, Spain; 4 Psychiatry Service, Hospital Universitario Miguel Servet, 50009 Zaragoza, Spain; 5 Centre for Healthy Brain Ageing, School of Psychiatry, University of New South Wales Medicine, 2052 Randwick, Australia; d.lipnicki@unsw.edu.au; 6 Psychiatry Service, Hospital Clínico Universitario Lozano Blesa, 50009 Zaragoza, Spain; 7 Department of Medicine and Psychiatry, Universidad de Zaragoza, 50001 Zaragoza, Spain; 8 Department of Psychology and Sociology, Universidad de Zaragoza, 50001 Zaragoza, Spain

**Keywords:** risk index, dementia, psychopathological risk factors, ZARADEMP, competing risk

## Abstract

With the increasing size of the aging population, dementia risk reduction has become a main public health concern. Dementia risk models or indices may help to identify individuals in the community at high risk to develop dementia. We have aimed to develop a novel dementia risk index focused on the late-life (65 years or more) population, that addresses risk factors for Alzheimer’s disease (AD) easily identifiable at primary care settings. These risk factors include some shown to be associated with the risk of AD but not featured in existing indices, such as hearing loss and anxiety. Our index is also the first to account for the competing risk of death. The Zaragoza Dementia and Depression Project (ZARADEMP) Alzheimer Dementia Risk Score predicts an individual´s risk of developing AD within 5 years. The probability of late onset AD significantly increases in those with risk scores between 21 and 28 and, furthermore, is almost 4-fold higher for those with risk scores of 29 or higher. Our index may provide a practical instrument to identify subjects at high risk of AD and to design preventive strategies targeting the contributing risk factors.

## 1. Introduction

There were 50 million people worldwide living with dementia in 2018, at an estimated financial cost to society of $1 billion. With the rapidly growing global population of older individuals, the prevalence of dementia and its associated cost are expected to double by 2030 [1]. Accordingly, dementia is recognized as a Public Health Priority by the World Health Organization (WHO) [2] and dementia risk reduction is one of the main targets in the Global Action Plan on the Public Health Response to Dementia 2017–2025 [3]. The most common form of dementia is Alzheimer´s Disease (AD), which may contribute to 60–70% of cases [4].

Given the considerable implications of dementia for affected individuals, their families and society, as well as there being no effective treatment, a preventive approach is crucial. Prevention strategies could both delay the onset of dementia and reduce its prevalence [5]. However, to optimize the effectiveness of risk reduction programs, it is necessary to know the modifiable risk factors for dementia and have reliable estimates of their effect size [6]. This can facilitate the development of a dementia risk score to identify individuals at high risk of developing dementia for targeting with prevention strategies.

Several models for predicting dementia have been developed (see Tang et al. [7] for a review). The CAIDE (Cardiovascular Risk Factors, Aging and Dementia) study developed a risk score for predicting late-life dementia in middle-aged people [8], but the model showed poor transportability to older aged cohorts [7] and low and middle income countries [9]. Other models have included risk variables that can be expensive to assess and are not universally available, such as brain imaging [10] or Apo-E4 genotype [11]. More recently developed risk models have reduced complexity [12] and incorporate self-reported variables, such as the Australian National University Alzheimer´s Disease Risk Index (ANU-ADRI) [13] and the LIfestyle for BRAin health (LIBRA) index [14], or include variables easily accessible in primary care, such as the Brief Dementia Screening Indicator (BDSI) [15] and the Framingham Heart Study risk score [16].

An external validation study of four dementia prediction models (including the CAIDE, ANU-ADRI and BDSI) in an elderly, community-dwelling sample found varying accuracies for predicting dementia (C-statistics and 95%CI at 5-year follow-up: CAIDE 0.54 (0.50–0.58), BDSI 0.80 (0.76–0.84), ANU-ADRI 0.78 (0.76–0.81) and DRS 0.82 (0.78–0.86)), as well as all models performing similarly to predictions based on age alone [17]. The authors recommended that new or refined dementia prediction models are needed. Dementia prediction models may be made more accurate by including risk factors not featured previously. To this end, depression and anxiety have been recently recognized as potentially useful [18]. Few dementia risk scores developed from population-based cohorts have included depression [13,14,15], and these used self-reported items or symptomatic scales rather than more stringent clinical criteria for depression known to have a higher association with dementia risk [19]. Systematic reviews and meta-analysis support anxiety as a major risk factor for dementia [20,21] and AD [22]. Furthermore, we have recently reported that clinically relevant anxiety is a risk factor for overall dementia [23] and AD [24] in the elderly. However, to the best of our knowledge, no dementia risk model has yet included anxiety. Hearing loss is another risk factor for dementia, as identified by the 2020 Lancet Commission report [25], which has not been included in previous dementia risk scores.

Death is a competing risk for the development of numerous diseases in old age, and it is recommended this mortality effect be considered in incidence studies [26]. While risk models for diabetes and coronary artery disease have used a competing risk analysis model in the presence of death [26,27], this does not appear to have been done for dementia. It has been acknowledged that not doing so might have biased the dementia risk predictions of previous models [14,17], and that future models should take the mortality effect into account [17].

In this study, we aimed to develop a new dementia risk score that includes depression, anxiety, and hearing loss in addition to the risk factors more commonly used. We intended for all included factors to be self-reportable or accessible to primary care doctors. Our model is further distinguished by taking the competing risk of death into account.

## 2. Materials and Methods

This work follows Strengthening the Reporting of Observational Studies in Epidemiology (STROBE) [28] and the Statistical Analysesand Methods in the Published Literature (SAMPL) [29] guidelines for reporting observational studies in epidemiology and statistics, respectively.

### 2.1. Sample and Procedure

We used data from the Zaragoza Dementia and Depression (ZARADEMP) Project, a longitudinal, population-based study intended to document the incidence and risk factors for somatic and psychiatric diseases, specifically dementia, in adults aged ≥55 years. The Ethics Committee of the Institutional Review Board (CEICA) approved the study, and principles of written informed consent, privacy, and confidentiality have been maintained throughout.

Wave I (ZARADEMP I) is a baseline, cross-sectional study, intended to identify a cohort of individuals without dementia, as well as the prevalence and distribution of the hypothesized risk factors for dementia. The field work for this baseline study was completed by well-trained lay interviewers (senior medical students). Interviews were conducted at participants home or, in the case of institutionalized subjects, at institution. The interviews lasted 25–90 min and incorporated validated Spanish versions of the following international assessment instruments: the Mini-Mental Status Examination (MMSE); Geriatric Mental State B (GMS-B); Automated Geriatric Examination for Computer-Assisted Taxonomy (AGECAT); History and Aetiology Schedule (HAS); Katz’s Index for basic activities of daily living (ADL), the Lawton and Brody scale for instrumental ADL, and the European Studies of Dementia (EURODEM) Risk Factors Questionnaire. The blood pressure, height and weight of each individual were also checked and recorded routinely in this phase of the study. Medical reports were used in some cases when available to help in the diagnostic process [30,31].

The Geriatric Mental State (GMS) is a well-known semistructured standardized clinical interview for assessing the mental state of elderly persons. It is also a syndrome case finding instrument for community subjects, and the computerized program AGECAT can be applied with this purpose [32]. The History and Aetiology Schedule (HAS) is a standardized method of collecting history and aetiology data from an informant, or directly from the respondent when they are judged to be reliable. It concentrates on those features expected to be relevant to psychiatric diagnosis in older people and is crucial to complete the GMS and facilitate a diagnostic process. The Risk Factors Questionnaire used in this study was designed by the EURODEM Workgroup [33]. The instrument is intended to include information related to the following potential risk factors of dementia, Azheimer’s Disease and vascular dementia: history of medical diseases, including cardiovascular disease, traumatic brain injury, epilepsy, Down’s Syndrome, Parkinson’s disease, diabetes mellitus, thyroid disease, abuse of alcohol or smoking; menopause; psychiatric history, in particular depression; use of medications; history of general. Each item in the interview has been operationally defined, according to previously agreed EURODEM criteria [30,31]. Individuals were nominated as “probable cases” on the basis of GMS threshold “global” scores (1/2) and/or MMSE standard cut-off points (23/24), decided on the bases of adequate negative predictive value. However, the data on each elderly were thoroughly reviewed by the research psychiatrists supervising individually the lay interviewers. In the final step of Wave I, the psychiatrists recorded a diagnosis of “dementia”, “depression”, “cases”. “Subcases” of “dementia” were also nominated on the basis of borderline scores on the same instruments. For a diagnosis of dementia, documented deterioration in ADL due to cognitive deterioration was required [30,31].

In the ZARADEMP study, the representative sample was drawn from Spanish official census lists, stratified with proportional allocation by age and sex, and included institutionalized individuals. The baseline assessment in 1994 included 4803 individuals. Here, we report results from baseline (Wave I) and two follow-up waves (Waves II and III). As we were interested in cognitively intact individuals, we excluded subjects considered to be cases or subcases of dementia at baseline (*n* = 746) for the follow-up, resulting in an initial sample of 4057 participants, of which 2704 participated in Wave II and then 2258 in Wave III. For an easy comparison with the existing literature, we have selected the age group of over 65 years (*n* = 3044).

### 2.2. Diagnosis of Incident AD at Waves II and III

A two-phase, screening procedure was implemented in each of the waves, II and III, using the Spanish versions of the international assessment instruments defined for Wave I. Participants were classified in phase I as “probable cases” of dementia based on the GMS threshold “global” score (1/2) and/or MMSE standard cut-off point (23/24). In phase II, all probable cases of dementia were reassessed in their place of residence by a research psychiatrist using the same instruments in phase I, as well as Hachinski’s scale [34], and a brief, previously standardized neurological examination. Incident dementia and type (AD, vascular, other) were initially diagnosed by the research psychiatrist, but a final diagnosis based on the Diagnostic and Statistical Manual of Mental Disorders-IV (DSM-IV) was made by a consensus panel (at least three of four psychiatrists in agreement). Our previous studies have supported the validity of this diagnostic process [35]. Moreover, to document the accuracy of the panel, a proportion of cases were invited for a hospital diagnostic work-up, which incorporated a neuropsychological battery and neuroimaging, and the National Institute of Neurological and Communicative Disorders and the Alzheimer`s Disease and Related Disorders Association (NINCDS-ADRDA) [36] criteria used to diagnose AD. Agreement between panel members on the diagnosis of dementia and AD was reached in 95.8% and 86.7% of cases, respectively [31].

### 2.3. Risk Factors Assessed

The potential risk factors were evaluated at baseline by lay-interviewers (individually supervised by research psychiatrist), and are classified as socio-demographic (age, sex, educational level, and marital status), psychological (anxiety and depression), behavioral (tobacco and alcohol use, and obesity), and medical (hearing loss, hypertension, diabetes, and history of angina, acute myocardial infarction or stroke).

#### 2.3.1. Socio-Demographic Risk Factors

To simplify the scoring method, continuous variables were categorized. For comparison with previous risk scores, we only include individuals aged 65 or more years, and categorized ages into three groups: 65–74, 75–84 and 85+ years. Educational status was classified as: “illiterate (unable to read and write, or with less than 2 years of formal education)”, “primary studies (complete or incomplete)” and “secondary education or above”. Marital status was defined as “single”, “married or living as a couple” and “formerly married (divorced, separated or widowed)”.

#### 2.3.2. Psychological Risk Factors

The diagnosis of anxiety and depression was based on the GMS-AGECAT system. After symptom assessment by the GMS-B Scale, a computer program compared syndrome clusters (e.g., dementia, depression, anxiety) to reach a final diagnosis, for this study we considered “case” levels of anxiety or depression (confidence levels ≥3). The recommended cut-off ≥3 has shown a good sensitivity (0.91) and specificity (0.89) for diagnosis of depression clinically significant; that means cases with current signs and symptoms of depression rated by clinicians severe enough to require antidepressant intervention [37].

#### 2.3.3. Behavioral Risk Factors

Alcohol and tobacco use were self-reported, and both categorized as user, non-user, or former user. Body mass index (BMI) was calculated as weight in kilograms divided by height in meters squared. A BMI greater than 30 kg/m^2^ was defined as “obesity”.

#### 2.3.4. Medical Risk Factors

Hearing loss was scored when the subject was almost or completely deaf. History of cardiovascular risk factors (angina, myocardial infarction or stroke) and diabetes was based on data from the EURODEM questionnaire [33]. A positive history of diabetes was based on a previous medical diagnosis and/or receiving treatment for diabetes. Blood pressure (BP) was calculated as the mean value of 2 measurements using a standard sphygmomanometer. Hypertension was defined as a systolic BP greater than 140 mmHg, a diastolic BP greater than 90 mmHg, and/or use of blood pressure-lowering drugs.

### 2.4. Statistical Analysis

We assessed dementia risk on cognitively intact subjects at baseline, according to risk of developing dementia at follow-up for every studied risk factor.

The follow-up period was the time from baseline (Wave I) to whichever of the following occurred first: dementia, death, loss to follow-up, or the end of follow-up (Wave III) after nearly 5 years. We used a competitive risk regression [38] or subdistribution hazards regression to adjust estimates of incident dementia risk taking into account the competitive mortality risk [39] conveyed by the overall duration of follow-up. The subdistribution hazard ratio (SHR) for assessing the risk of developing dementia for an individual factor was calculated using the *cmprsk* library in the *R* package, which allows adjustment for socio-demographic and clinical variables. The SHR is a way of expressing the instantaneous risk of developing a given event in an individual who has not yet experienced such an event (at risk), when taking into account a competing risk, such as death [40]. A higher SHR would mean a higher risk of developing AD for this factor, taking into account death as a competing risk. 

We chose an approach similar to that of Li et al. [16]. First, all potential risk factors were included separately in subdistribution hazards regression models. Factors that reached *p* ≤ 0.1 were then simultaneously included in a multivariate prediction model and as variables of the risk score. For each model, the SHR and 95% confidence interval were computed. To examine the proportional hazards assumption, the time-varying effect of each covariate was tested using the Scheike and Zhang test [41].

Since all variables were categorical, their estimated contribution to the risk of dementia could be expressed by simplified scores assigned to each category. We assigned a risk score for each factor using the β-coefficients of multivariate subdistribution hazard models. To facilitate interpretation, β coefficients were standardized by dividing them by the lowest observed value (i.e., the lowest then had a value of 1) and rounding to the closest integer. Since the lowest β value was 0.15 and its multiplication by 6.7 makes it approximately 1, all β values were multiplied by 6.7 and were rounded to the closest integer [8]. For additional analyses, we summed these scores as predictors of the risk of AD incidence over a 5-year follow-up period. This was performed for each participant based on a cumulative incidence function (CIF), taking into account the competing event (death) as time progressed [42], as we have previously done [31].

## 3. Results

Our final sample included 3044 participants aged 65+ without dementia at baseline (median 4.4 years; interquartile range: 2.9–4.9 years). During the follow-up period, 663 (21.8%) individuals died, 582 (19.1%) were lost (by refusal to take part, changing residence or being impossible to contact), 85 (2.8%) were incident AD cases and 47 (1.5%) were incident cases of other dementias. Using a competitive risk regression model, they were all included in the risk calculation [38].

Table 1 shows baseline demographic characteristics according to AD incidence status. Participants with incident AD were significantly older, more likely to be female, formerly married, and to have lower educational level and higher anxiety than participants without AD.

Of the risk factors assessed, those associated with AD risk in univariate regression models were age, sex, education, marital status, anxiety, BMI, and hearing loss (Table 1). These factors were included in a multivariate model, with Table 2 showing the β-coefficients and SHRs for AD incident cases and the risk scores assigned to each risk factor. Depression was not significantly associated with AD risk, but was kept in the final model because we previously found in a meta-analytic study including our sample that participants with clinically significant depression had a two-fold higher risk of AD [43] and that severe depression was associated with a 4-fold risk of incident AD in our sample [44]. In addition, removal of depression from the analysis did not change the scores derived for the other factors.

Based on selected factors, the total score ranged from 0 to 56 (Table 2). For each one-point increment in the risk scale, the AD risk increased significantly by 16% (SHR: 1.16; 95% CI: 1.12–1.19; *p* < 0.001). Table 3 shows that the risk of AD increased across risk score categories, defined by dividing our sample into quartiles. Using our risk scale, an 85-year-old male, with higher education, single, without depression or anxiety, overweight and without hearing loss has 17 risk points in total and a 1.78-fold risk of AD. However, an 85-year-old woman, with primary education, married, anxiety, depression, normal weight, and hearing loss has 48 risk points and a 22.6-fold risk of AD. Finally, Table 4 shows the score sheet developed to predict Alzheimer’s disease.

## 4. Discussion

The “ZARADEMP Alzheimer Dementia Risk Score” predicts an individual’s risk of developing AD within 5 years based on selected risk factors easily accessible in primary care settings: age, sex, education, marital status, depression, anxiety, BMI, and hearing loss. Most variables are assessed by direct questions, and obesity and clinically significant anxiety and depression are regularly approached by primary care doctors in their daily routine practice. Our index could be easily applied by any clinician aiming to assess the risk of AD in their routinely practice (primary care doctors, neurologist, psychiatrist, clinical psychologist, or geriatrist). Moreover, risk index could be calculated in a simple way: the specific risk scores (RS) for any of the variables are summed up for each individual. For example, a 76-year-old (RS 11) woman (RS 3), widowed (RS 11), with primary studies (RS 2), with clinically significant anxiety (RS 8) but not clinically depressed (RS 0), non-obese (RS 4), and without hearing loss (RS 0), would have a total risk score of 39 for developing AD at 5 years of follow-up. The probability of late-onset AD was significantly high for risk scores between 21 and 28, but almost 4-fold higher than this for risk scores 29+. 

In our final model, age was the factor most strongly associated with AD risk, as expected, since age is consistently the greatest risk factor for overall dementia [25]. Prevalence of AD increases continuously and exponentially with age, being reported 3% in subjects from 65 to 69 years old and 32% at age of 85 or older [45]. While previous studies have consistently shown women as having higher prevalence of AD than men, results about differences in the risk of developing AD for men and women of the same age are mixed [45]. Consistently, women in our sample showed a significant increased risk of AD related to men in the univariate regression model, but results were not statistically significant in the multivariate regression model. Nonetheless, a tendency for increased risk of AD in women was observed, and this variable represents 3 points in our final risk score. The greater risk of AD associated with illiteracy is consistent with previous results that suggest an inverse association between educational achievement and risk of dementia, [46,47] supporting the construct of “cognitive reserve”. “Cognitive reserve”, or “reserve” [48], refers to the brain´s ability to develop cognitive networks that enable a person to continue to perform cognitive task despite degenerative brain changes [45,48]. Besides years of formal education, or even genetic or other environmental factors [48], engaging in stimulating mental activities may also help to build cognitive reserve [45], so that this could be a modifiable factor of AD. As to civil status, individuals formerly married had a higher risk of AD than single or married individuals, maybe because of greater loneliness [49] which has been shown to contribute to dementia in a previous meta-analysis [50]. In line with the literature, we found a higher incidence of AD in subjects with hearing loss. It has been widely associated in the literature with the risk of AD [51] and all-cause dementia [52], and several hypotheses about such causal relationship have been proposed. Among them, it is hypothesized that it could lead to social isolation, and this to dementia [51]. In addition, it has been shown that the genetic risk of AD also influences the hearing of speech in noise, without these hearing deficits being related to further cognitive impairment [53]. Regarding obesity, we found in our elderly sample that higher BMI had protective effects on dementia risk. However, obesity in midlife has been identified as a risk factor for dementia [25]. Our results are consistent with those of Li et al. [16], and support previous studies that found age-dependent effects of obesity on dementia risk [54]. Similar age-dependent effects have been described for hypertension [55]. It would be interesting to study the effect of other behavioral factors such as the habitual consumption of specific products, as effects on memory have been observed [56] and as this should be easily available information in a primary care interview. Interestingly, we found that clinically significant anxiety showed a much stronger association with AD risk than clinically significant depression, and that these psychological variables contributed to AD risk more than cardio-vascular risk factors and diabetes.

Compared to previous indices, our “ZARADEMP Alzheimer Dementia Risk Score” includes mainly socio-demographical and psychological variables and is the first to include hearing loss and anxiety. Notice that anxiety had a moderate-high weight on total risk score (8 points) and contributes to AD risk as much as illiteracy. Moreover, we assessed current and clinically significant anxiety and depression, whereas previous indices assessed depression using self-report symptomatic scales. A large meta-analysis of dementia risk estimates for depression [19] demonstrated higher risk estimates for depression assessed by more stringent, and previously validated against clinical criteria than those using a milder cut-off in symptomatic scales. In this sense, depression according to GMS-AGECAT criteria has shown an acceptable overall agreement with depression according to DSM criteria and higher sensitivity to detect clinically significant depression in elder people [37]. While we did not find clinically significant depression to be significantly associated with the risk of AD in our sample, we decided to include it in our index because of depression being a well-established risk factor for dementia and AD [19,25] and previously reported associations based on [44] or including our sample [43]. We previously found significant results in our sample only for severe depression [44], but in a further meta-analysis, with more power to detect an effect than our individual study, we found significant results for clinically significant depression [43]. However, depression only added 1 point to our risk score.

Some of the risk factors included in our index, such as anxiety and depression, might not be independent of AD and could be prodromal symptoms of the disease prior to clinical diagnosis. Despite the exclusion of subjects with cognitive impairment, the lack of biomarkers for AD in our sample could have led to preclinical AD being underestimated. However, we assessed clinically significant depression and anxiety, excluding mild/ subsyndromal symptoms, and we think that the higher specificity in diagnosis of depression and anxiety could support the hypothesis of the real risk interpretation, as opposed to prodromal symptoms of emotional dysregulation described as part of the Mild Behavioral Impairment construct [57]. This is currently a controversial question and further studies are needed, because the few studies that have explored longitudinal trajectories of depression in preclinical phase of dementia [58,59] suggest that depression is more likely to be a prodromal symptom and related to dementia-related brain changes.

In addition, a recent systematic review states that subpopulations with different risk profiles need to be considered and tailored scales created [60]. In this sense, it has been observed that previous models carried out on middle-aged cohorts have shown poor transferability to older cohorts [7], making age-specific models such as ours highly relevant to improve dementia prediction.

### Strengths and Limitations

The main highlights of our index are that it includes anxiety as a modifiable risk factor for AD and accounts for competing risk of death for the first time. Previous indices did not control for mortality, and survival bias may have affected their results.

Our study includes relevant variables derived from meta-analyses of cohort studies reporting risk factors for dementia and AD [25]. However, other potential risk factors for dementia, such as physical inactivity [25] or cognitive engagement [61], were not assessed in the ZARADEMP study and, therefore, were not included in our index. The variable “living alone”, which has been shown to be associated with an increased risk of dementia [62], was not analyzed separately from marital status. The use of hearing aids, which have been shown to delay diagnosis of dementia and AD in individuals with hearing loss [63], was also not specifically collected in our study.

The factors included in our risk score are based on results from a representative, large sample of the general population older than 65 years, excluding participants with dementia at baseline. However, being based on a single cohort limits the generalizability of our index [14]. The applicability could also be limited if using the index to predict AD risk in late life (older than 65) or in a relatively short term (5 years). Our model could not be applied at earlier ages and for longer follow up, this is a limitation because the brain changes in dementia can start up to 10 years before the initial symptoms appear. Further validation of the “ZARADEMP Alzheimer Dementia risk score” is required.

## 5. Conclusions

The “ZARADEMP Alzheimer Dementia Risk Score” may increase our understanding of the weight that specific risk factors, most of them modifiable, have on AD burden at the population level. Our risk score includes, for the first time, current, clinically significant anxiety and the variable hearing loss. Moreover, it may provide a practical instrument to identify subjects at high risk in routine primary care practice, and towards whom preventive strategies targeting the contributing factors could be directed.

As the goal of the current work was to describe variables included in our new dementia risk index, further validation to assess applicability and generalizability of our score is required, as well as further studies that compare the performance of our risk scores with those previously developed by other authors.

## Figures and Tables

**Table 1:** Baseline characteristics according to incident AD status and associations between individual risk factors and AD risk.

Variables	Follow-Up AD Status			Univariate Regression Model	
	No AD (*N* = 2959)	Incident AD (*N* = 85)	*p*-Value	SHR (95% CI) ^a^	*p*-Value
Sociodemographic characteristics					
Age (years)	75.4 (7.7)	84.2 (6.3)	<0.001	1.14 (1.12–1.17)	<0.001
Female sex	1645 (55.6%)	59 (69.4%)	0.016	1.81 (1.14–2.87)	0.012
Education (years)	7.3 (3.8)	5.9 (3.8)	0.001	0.89 (0.82–0.96)	0.003
Marital status (ref. single)			<0.001		
Married/in couple	1690 (57.1%)	25 (29.4%)		2.06 (0.49–8.68)	0.320
Formerly married	980 (33.1%)	58 (68.2%)		8.18 (2.00–33.39)	0.003
Psychological risk factors					
Depression	306 (10.3%)	14 (16.4%)	0.101	1.44 (0.81–2.55)	0.210
Anxiety	66 (2.2%)	6 (7.0%)	0.011	3.28 (1.43–7.54)	0.005
Behavioral risk factors					
Alcohol or Smoking	758 (25.6%)	20 (23.5%)	0.757	0.90 (0.54–1.49)	0.680
BMI	26.9 (7.3)	26.3 (5.3)	0.463	0.63 (0.44–0.90)	0.012
Medical risk factors					
Diabetes	395 (13.5%)	10 (11.9%)	0.803	0.87 (0.45–1.68)	0.680
Hypertension	2112 (71.5%)	55 (64.7%)	0.214	0.73 (0.47–1.15)	0.170
Hearing loss	22 (0.7%)	2 (2.3%)	0.304	3.17 (0.80–13.20)	0.022
Angina	177 (6.1%)	4 (4.7%)	0.776	0.75 (0.27–2.04)	0.570
Myocardial infarction	86 (3.0%)	4 (4.8%)	0.527	1.61 (0.59–4.38)	0.350
Stroke	171 (5.8%)	5 (5.9%)	0.845	1.04 (0.80–2.57)	0.930

Notes: Data are given as mean (standard deviation) or number (%); AD: Alzheimer’s disease; CI: confidence interval; SHR: subdistribution hazard ratio. ^a^ Reported SHR of AD is related to non-cases, CIs and *p* values related to SHR were from “normal approximation” of Wald χ^2^ test with 1 *df*.

**Table 2:** Risk factor associations with AD risk in a multivariate regression model.

Variables	β Coefficient	SHR (95% CI) ^a^	*p*-Value	Risk Score
Sociodemographic characteristics				
Age				
65–74 (*n* = 1584)	0 (reference)	1 (reference)		0
75–84 (*n* = 902)	1.64	5.16 (2.32–11.50)	<0.001	11
Over 85 (*n* = 558)	2.54	12.66 (5.71–28.06)	<0.001	17
Sex				
Male (*n* = 1340)	0 (reference)	1 (reference)		0
Female (*n* = 1704)	0.46	1.59 (0.92–2.74)	0.096	3
Education (years)				
Secondary or higher (*n* = 479)	0 (reference)	1 (reference)		0
Primary (*n* = 2275)	0.23	1.26 (0.59–2.71)	0.550	2
Illiterate (*n* = 266)	1.08	2.95 (1.23–7.10)	0.015	8
Marital status				
Single (*n* = 284)	0 (reference)	1 (reference)		0
Married/in couple (*n* = 1715)	1.26	3.51 (0.80–15.41)	0.096	9
Formerly married (*n* = 1038)	1.66	5.28 (1.27–22.00)	0.022	11
Psychological risk factors				
Depression				
No case (*n* = 2674)	0 (reference)	1 (reference)		0
Case (*n* = 370)	0.15	1.16 (0.64–2.12)	0.630	1
Anxiety				
No case (*n* = 2972)	0 (reference)	1 (reference)		0
Case (*n* = 72)	1.20	3.32 (1.39–7.94)	0.007	8
Behavioral risk factors				
BMI				
Overweight/Obesity (*n* = 2051)	0 (reference)	1 (reference)		0
Normal (*n* = 993)	0.50	1.65 (1.04–2.63)	0.034	4
Medical risk factors				
Hearing loss				
No case (*n* = 3014)	0 (reference)	1 (reference)		0
Case (*n* = 24)	0.49	1.63 (0.35–7.47)	0.530	4

Notes: AD: Alzheimer’s disease; CI: confidence interval; SHR: subdistribution hazard ratio.^a^ Reported SHR of AD is related to non-cases, CIs and *p* values related to SHR were from “normal approximation” of Wald χ^2^ test with 1 *df*.

**Table 3:** Fine and Gray regression model relating the score quartiles with risk of AD.

Risk Score	Univariate Regression Model			
	No. at Risk ^a^	Incident AD Cases (%)	SHR (95% CI) ^b^	*p*-Value
0–14	954	4 (0.4%)	1 (reference)	
15–20	676	5 (0.7%)	1.78 (0.48–6.63)	0.390
21–28	714	17 (2.4%)	5.78 (1.95–17.16)	0.002
29+	663	59 (8.9%)	22.61 (8.23–62.12)	<0.001

Notes: AD: Alzheimer’s disease; CI: confidence interval; SHR: subdistribution hazard ratio. ^a^ Of the 3044 participants in the baseline, 27 had missing risk score values and were excluded, leaving a total of 3007 at risk. ^b^ Reported SHR of AD is related to non-cases, CIs and *p* values related to SHR were from “normal approximation” of Wald χ^2^ test with 1 *df*.

**Table 4:** Score ranges and probability of AD within 5 years for individuals aged over 65 years.

Risk Score	5 yr AD Probability (%)
0–5	0.11
6–10	0.24
11–15	0.49
16–20	1.02
21–25	2.13
26–30	4.41
31–35	9.01
36–40	17.92
41–45	33.82
46–50	57.81
50+	83.54

Notes: AD: Alzheimer’s disease; yr: year.
